# The noncoding RNA GRAS1 interacts with NKAP and protects against DNA damage and cell death

**DOI:** 10.26508/lsa.202403105

**Published:** 2026-09-30

**Authors:** Tong Su, Nhu Trang, Lingbo Kong, Darin Cheung, Jonathan Zhu, Vita Chou, Lauren Ellis, Nichelle Camden, Calvin Huang, Colleen A McHugh

**Affiliations:** Department of Chemistry and Biochemistry, University of California San Diego , La Jolla, CA, USA

## Abstract

Noncoding RNA (ncRNA) gene products may be involved in diverse biological processes. We find that the growth regulator antisense 1 (GRAS1) noncoding RNA (ncRNA) transcript protects against cell death in human cell lines. GRAS1 ncRNA is one of the biomolecules produced from the *EPB41L4A-AS1* gene locus, which also encodes the SNORA13 snoRNA and the TIGA1 protein. Specific knockdown of GRAS1 ncRNA leads to cell death and extensive DNA damage. We used RNA antisense purification and mass spectrometry to identify NF-κB activating protein (NKAP) as a direct interaction partner of GRAS1 ncRNA. NKAP was degraded in a proteasome-dependent manner after GRAS1 knockdown. Complementation with overexpressed NKAP mitigated the DNA damage effects of GRAS1 knockdown and confirmed the contribution of both NKAP and GRAS1 to cell growth. In summary, we find that GRAS1 and NKAP directly interact and protect against DNA damage and cell death in lung cancer and colon cancer cell lines. Our identification of a functional GRAS1 transcript reveals new information about the biomolecules produced by the *EPB41L4A-AS1* locus and their contributions to human cell growth.

## Abstract (precis)

This study uses a combination of molecular biochemistry and cell biology approaches to identify a novel function for a noncoding RNA and protein interaction in promoting human cell survival.

## Introduction

Noncoding RNAs (ncRNAs) are a large class of biomolecules with the potential to regulate cell growth processes. Dysregulated ncRNA expression is associated with cancer development and metastasis (Kazemzadeh et al, 2015). Direct binding interactions between noncoding RNA and proteins can regulate critical biological processes in humans (Wang & Chang, 2011). For example, XIST RNA coordinates X chromosome silencing. To initiate this process, XIST binds and recruits the transcriptional regulator SPEN/SHARP (McHugh et al, 2015). Most noncoding RNA functions remain unknown in human cells. Here, we examined the mechanism of the growth regulator antisense 1 (GRAS1) ncRNA in promoting cell growth.

GRAS1 RNA is a spliced noncoding transcript produced by the *EPB41L4A-AS1* gene locus. This locus was previously identified as a growth regulator in multiple human cell types in a CRISPR/Cas9 screen (Liu et al, 2017). Based on bioinformatic studies, altered expression of the *EPB41L4A-AS1* locus affects cell growth in a broad range of cancer types including gastric (Delshad et al, 2020), lung (Wang et al, 2020), colorectal (Bin et al, 2021), cervical (Liao et al, 2019), breast (Yang & Lv, 2022), placental villus (Zhu et al, 2019), and bone marrow–derived mesenchymal stem cells (Cui et al, 2020). The multifunctional *EPB41L4A-AS1 *locus produces at least three distinct biomolecules: Transcript Induced by Growth Arrest 1 (TIGA1) protein, an intronic small nucleolar RNA H/ACA Box 13 (SNORA13 snoRNA), and the GRAS1 noncoding RNA. The TIGA1 protein has been shown to be strongly up-regulated upon growth arrest, after at least 3 d of serum starvation in human fibroblasts (Yabuta et al, 2006). The SNORA13 snoRNA was previously demonstrated to be involved in cellular senescence by disrupting ribosomal biogenesis and activating p53 pathways in a transgenic human cell line model (Cheng et al, 2024).

In this study, we characterize the function of the GRAS1 ncRNA transcript and uncover a direct interaction between GRAS1 RNA and NKAP protein. NKAP was first studied in yeast (Chen et al, 2003) and has since been characterized as an RNA-binding protein in humans (Burgute et al, 2014). NKAP is a multifunctional protein, which is also required for accurate genome segregation in HeLa cells (Li et al, 2016). NKAP promotes growth and development in highly proliferative cell types (Pajerowski et al, 2009; Okuda et al, 2015; Shapiro et al, 2019). NKAP dysregulation has been associated with gastric cancer (Wei et al, 2019), colon cancer (Shu et al, 2019), glioblastoma (Sun et al, 2022), and renal cell carcinoma (Ma et al, 2020). There are no reported deletions or truncation mutants of NKAP in humans, suggesting that it is an essential protein. Missense mutations in the C-terminal region of NKAP cause Hackmann–Di Donato–type X-linked syndromic intellectual development disorder (MRXSHD), characterized by neurological and developmental delays in children (Fiordaliso et al, 2019). Here we investigated the molecular function of the GRAS1 RNA produced from the *EPB41L4A-AS1* locus and found that the interaction between GRAS1 and NKAP protects against DNA damage and promotes cell survival.

## Results

### *EPB41L4A-AS1* gene locus encodes GRAS1 noncoding RNA, SNORA13 small nucleolar RNA, and TIGA1 protein

**Figure 1.**
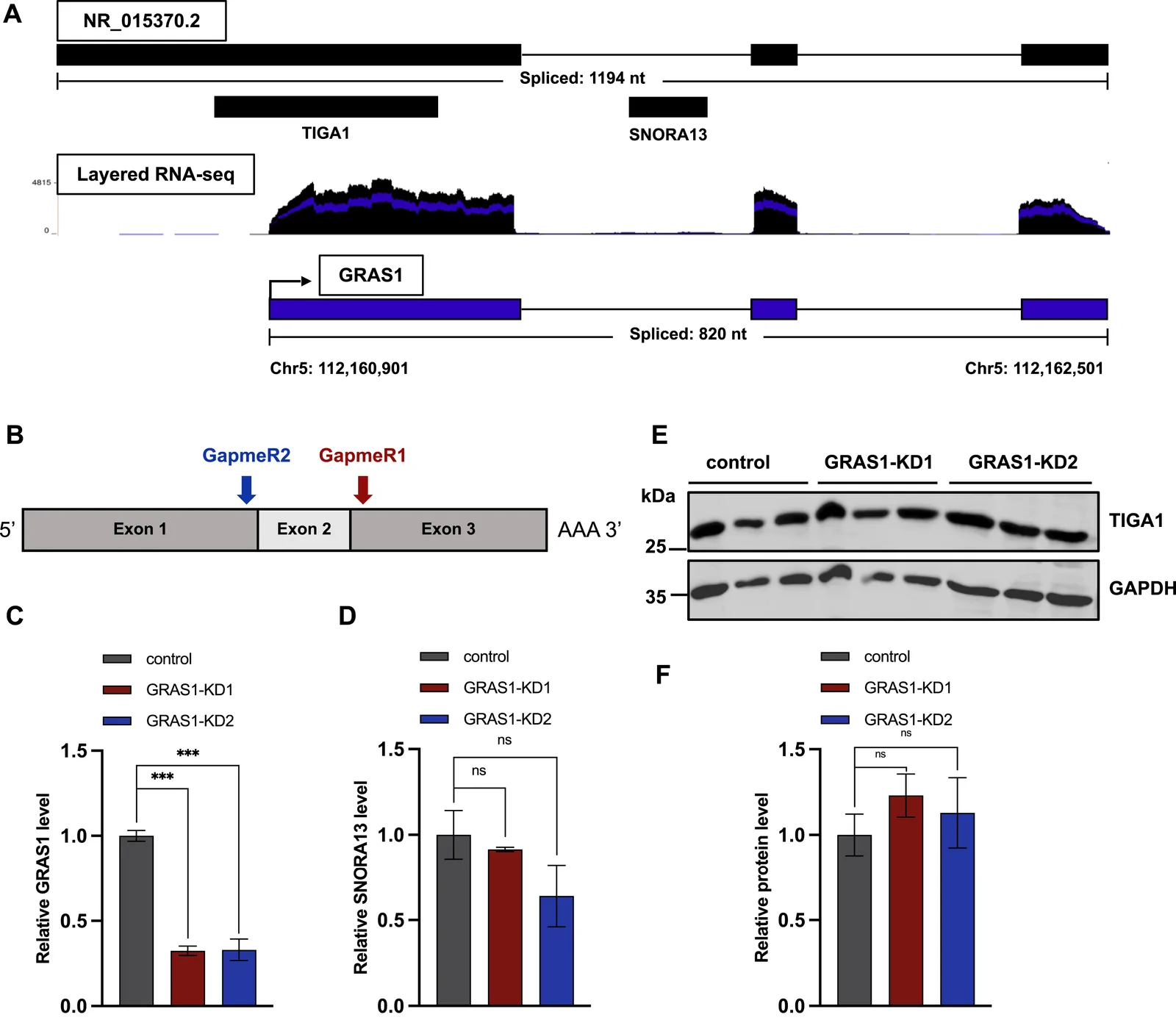
*EPB41L4A-AS1* gene locus encodes GRAS1 long noncoding RNA, SNORA13 small nucleolar RNA, and TIGA1 peptide. **(A)** GRAS1 long noncoding RNA locus and ENCODE RNA-seq expression data from the UCSC Genome Browser. **(B)** Schematic of ASO GapmeR1 and GapmeR2 targeting regions on GRAS1 gene. **(C)** RT-qPCR analysis of GRAS1 knockdown efficiency in A549 cells transfected with control GapmeR compared with GRAS1 GapmeR1 or GRAS1 GapmeR2, normalized to GAPDH control mRNA; N = 3. **(D)** RT-qPCR analysis of SNORA13 expression level in A549 cells transfected with control GapmeR compared with GRAS1 GapmeR1 or GRAS1 GapmeR2, normalized to GAPDH control mRNA; N = 3. **(E)** Immunoblot of TIGA1 in A549 cells treated with control GapmeR, GRAS1 GapmeR1, or GRAS1 GapmeR2. **(F)** Quantification of immunoblot (E). TIGA1 expression was calculated after normalization to the GAPDH loading control for each sample; N = 3. Error bars represent SD in all panels; (ns) *P* > 0.05; (***) *P* < 0.001 by two-tailed *t* test.

**Figure S1.**
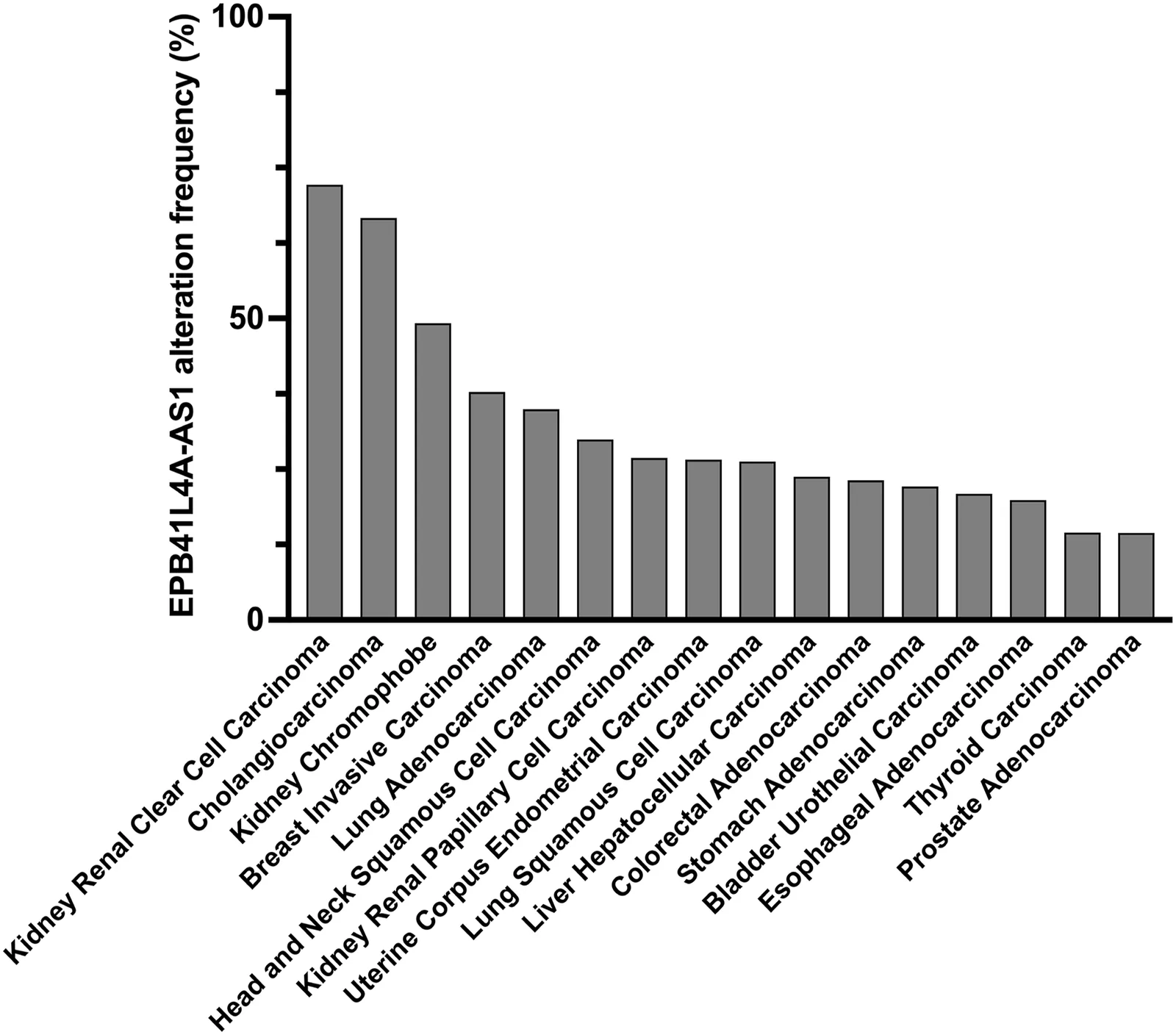
EPB41L4A-AS1 expression is dysregulated in cancer. TCGA PanCancer Atlas analysis of EPB41L4A-AS1 differential expression in tumors relative to adjacent normal tissues across 16 cancer types via cBioPortal. RNASeqV2 mRNA expression data were curated from 16 TCGA PanCancer Atlas Cohorts. Z-score threshold ± 2.

**Figure S2.**
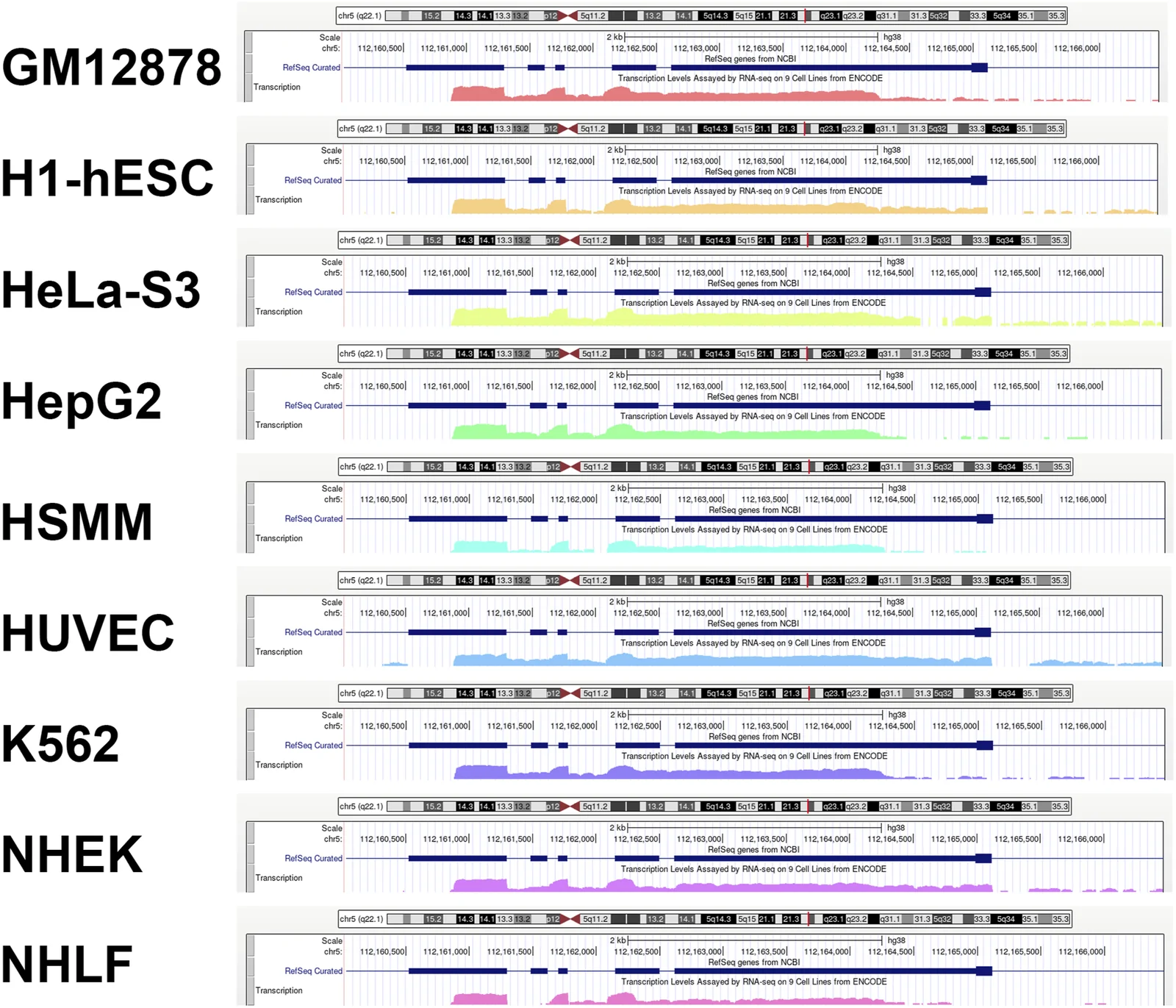
Individual cell type tracks for GRAS1 expression from EPB41L1A-AS1 locus in the ENCODE Integrated Regulation: Transcription RNA-seq Set. The cell line name is indicated on the left side of each expression track.

The GRAS1 ncRNA is located at Chromosome 5q22.1 in the *EPB41L4A-AS1 *locus. Consistent with previous reports, analysis of RNA expression data from The Cancer Genome PanCancer Atlas through cBioPortal showed that the *EPB41L4A-AS1* locus is altered in multiple cancer types, indicating a potential cancer-associated function of the gene locus (Figs 1A and S1; Cerami et al, 2012; Gao et al, 2013). The annotated noncoding RNA transcript associated with *EPB41L4A-AS1* locus is the NCBI Refseq transcript NR_015370.2. A detailed examination of RNA sequencing data at the *EPB41L4A-AS1* locus revealed that an unannotated RNA isoform is the predominantly expressed RNA transcript in the nine cell types that are included in the ENCODE Integrated Regulation: Transcription RNA-seq track set on the UCSC Genome Browser. We propose the name GRAS1 for the major unannotated RNA transcript produced from the *EPB41L4A-AS1* locus, which spans from Chr5:112,160,901 to 112,162,501 in the human GRCh38 genome assembly (Fig 1A). The GRAS1 RNA isoform is shorter than the NR_015370.2 sequence and differs at the 5′ end of the transcript. The GRAS1 transcript produces an 820-nucleotide spliced noncoding RNA. The 5′ end of the annotated NR_015370.2 gene is not significantly transcribed in the nine cell lines included in the ENCODE Integrated Regulation data set (GM12878, H1-hESC, HeLa-S3, HepG2, HSMM, HUVEC, K562, NHEK, NHLF) (Figs 1A and S2). The coding sequence for the TIGA1 protein is fully contained within the annotated NR_015370.2 transcript. The GRAS1 RNA transcript partially overlaps with the TIGA1 coding region at the *EPB41L4A-AS1* locus, but GRAS1 does not contain the translation start site for the TIGA1 protein and thus is not expected to encode the protein. Because the role of the GRAS1 noncoding RNA in cancer cell growth was not previously explored, we focused our analysis on the function of this transcript for the present study.

**Figure S3.**
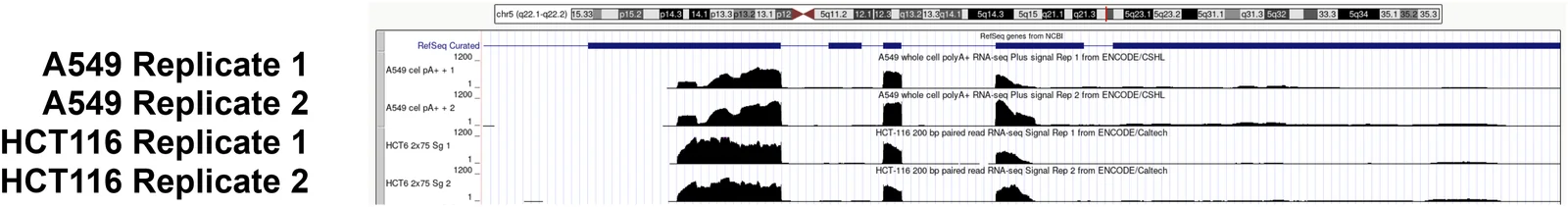
RNA-seq expression data at EPB41L1A-AS1 locus in A549 cells from ENCODE/Cold Spring Harbor (Gingeras Lab) and HCT116 cells from ENCODE/Caltech (Wold Lab). The cell line and replicate are indicated on the left side of each expression track.

The *EPB41L4A-AS1* gene locus was independently identified in prior CRISPR/Cas9 screens as a growth-promoting locus. Therefore, we tested the survival of human cells after knockdown of GRAS1 transcript with two specific antisense oligonucleotides (ASO) consisting of locked nucleic acid GapmeRs. In A549 cells, the major product of the *EPB41L4A-AS1 *locus is similar to datasets in the other cell types where we initially noted the unannotated GRAS1 transcript production from a downstream start site compared with the annotated NR_015370.2 transcript (Fig S3). We designed two independent ASO GapmeRs targeting different exons of the GRAS1 RNA transcript, without significant off-target binding to other transcripts in the human genome (Fig 1B) and tested the knockdown efficiency in non–small-cell lung cancer cells (A549). We initially focused on this cancer cell type for detailed analysis of GRAS1 RNA function because GRAS1 is highly expressed in this cell line, and because non–small-cell lung cancer is a leading cause of cancer morbidity and mortality in the United States, where identification of new molecular targets for potential therapeutic development is still needed. A reduction of around 60% of the GRAS1 RNA level in A549 cells was reproducibly achieved after treatment with either of the GRAS1-targeting ASO GapmeRs, compared with the non-targeting control GapmeR (Fig 1C). The ASO GapmeR knockdown method of GRAS1 manipulation was chosen, rather than deletion or mutation by CRISPR/Cas9 or other genetic alterations, to avoid changes to the DNA sequence that could affect the function of nearby enhancers or alter the transcriptional regulation of neighboring genes or the other products of the gene locus. ASO GapmeR targeting of RNA transcripts can be highly specific, has high bioavailability, and can achieve RNA target depletion in the nucleus (Maranon & Wilusz, 2020).

To confirm the specificity of GRAS1 ASO GapmeR knockdown for the GRAS1 ncRNA transcript and not the other gene products at this locus, we evaluated the cellular level of SNORA13 and TIGA1 after GRAS1 depletion by treatment with the two independent GapmeRs. GRAS1 knockdown with either of the two ASO GapmeRs did not change the expression of SNORA13, as measured by qRT-PCR (Fig 1D), or the protein level of TIGA1, as measured by immunoblotting of cell lysates (Fig 1E and F). This indicates that the ASO GapmeRs used for GRAS1 knockdown are highly specific to the GRAS1 ncRNA and allow the neighboring gene products to remain unaffected.

### GRAS1 knockdown causes cell death in multiple cell types

**Figure 2.**
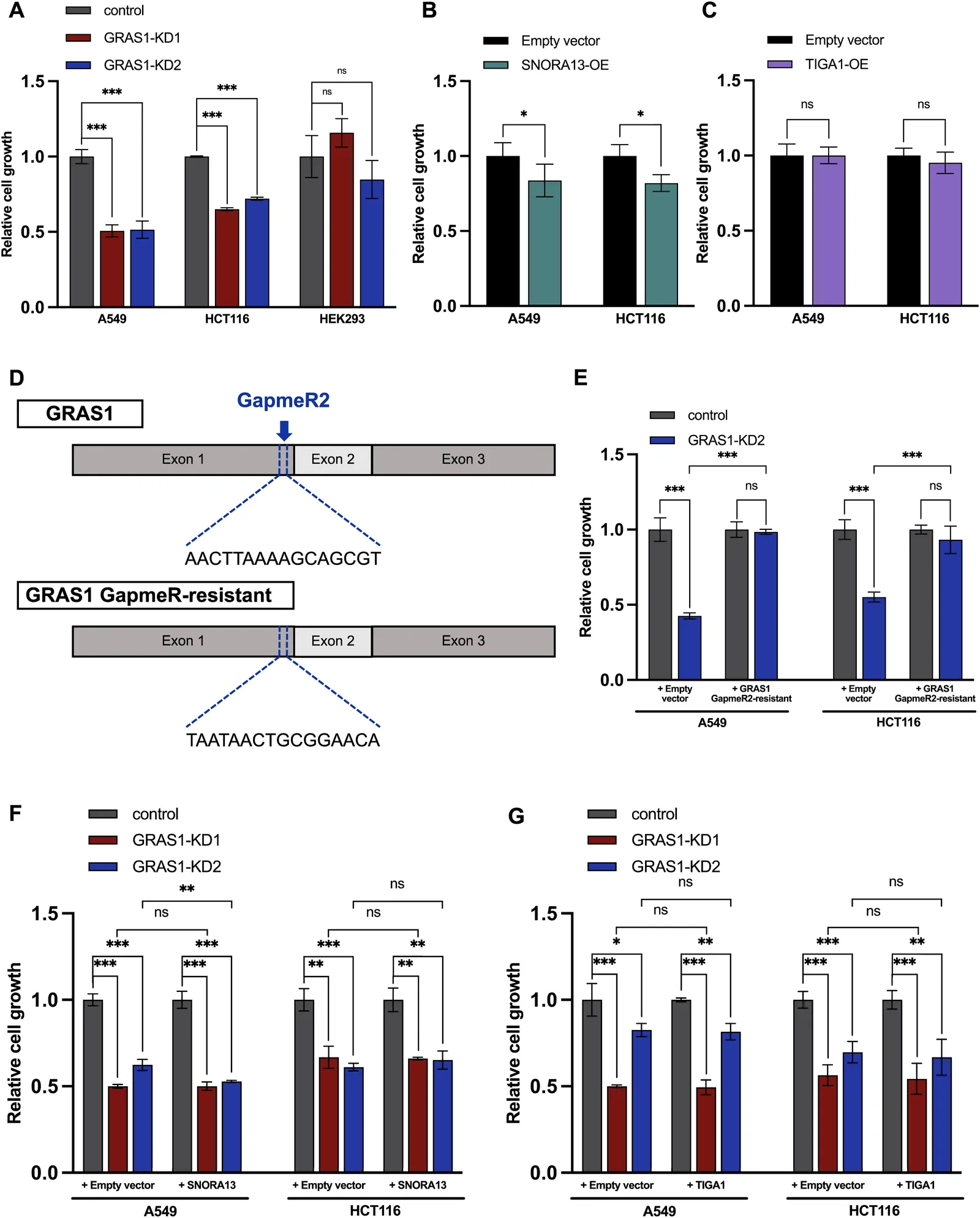
GRAS1 RNA knockdown causes cell death in multiple cell types. **(A)** CCK8 assay for survival of A549, HCT116, and HEK293 cells transfected with control GapmeR compared with GRAS1 GapmeR1 or GRAS1 GapmeR2; N = 3. **(B)** CCK8 assay for survival of A549 and HCT116 cells transfected with empty vector compared with SNORA13 vector; N = 4. **(C)** CCK8 assay for survival of A549 and HCT116 cells transfected with empty vector compared with TIGA1 vector; N = 4. **(D)** Schematic gene structure of GRAS1 GapmeR–resistant construct. **(E)** CCK8 assay for survival of A549 and HCT116 GRAS1 knockdown cells complemented with GRAS1 GapmeR–resistant vector compared with empty vector; N = 4. **(F)** CCK8 assay for survival of A549 and HCT116 GRAS1 knockdown cells complemented with SNORA13 vector compared with empty vector; N = 4. **(G)** CCK8 assay for survival of A549 and HCT116 GRAS1 knockdown cells complemented with TIGA1 vector compared with empty vector; N = 4. Error bars represent SD in all panels; (ns) *P* > 0.05; (*) *P* ≤ 0.05; (**) *P* ≤ 0.01; (***) *P* < 0.001 by two-tailed *t* test.

Next, we examined whether alteration of GRAS1 expression level affected survival in A549 cells and in other established human cell lines. We tested the growth effects of GRAS1 knockdown in cancer cell lines from two cancer types with the highest mortality rates in the United States, non–small-cell lung cancer (A549) and colorectal carcinoma (HCT116), along with the widely used human embryonic kidney cell line (HEK293). Treatment with either of the GRAS1 ASO GapmeRs dramatically decreased survival of A549 and HCT116 cells, as measured by mitochondrial function assay, whereas HEK293 cells remained unaffected (Fig 2A). In addition, we altered the cellular level of SNORA13 and TIGA1 with ectopic overexpression to evaluate the importance of these *EPB41L4A-AS1 *gene products in cell growth. In alignment with previous work, up-regulation of SNORA13 expression caused a reduction in cell survival in both A549 and HCT116 cells (Fig 2B). Increasing TIGA1 expression did not result in significant effects on cell survival under normal culture conditions in serum-replete medium (Fig 2C).

To further validate the observed phenotypes, GRAS1 knockdown cells were complemented by overexpression of a GRAS1 GapmeR–resistant construct through transient transfection. To create the GapmeR-resistant version of GRAS1 used for complementation studies, the GapmeR targeting sequence on the subcloned GRAS1 gene was randomly shuffled to prevent degradation of the overexpressed GRAS1 RNA by ASO GapmeR (Fig 2D). The impact of GRAS1 depletion on cell viability was rescued by complementation with the GapmeR-resistant GRAS1 construct in both A549 cells and HCT116 cells (Fig 2E). In addition, we attempted the rescue experiments by complementing GRAS1 knockdown cells with SNORA13 or TIGA1, and neither improved cell survival (Fig 2F and G). These results indicate that GRAS1 ncRNA contributes directly to cell survival, and the observed phenotypes are not due to the disruption of SNORA13 or TIGA1 in these cell lines.

### GRAS1 knockdown results in up-regulation of DNA damage response genes

**Figure 3.**
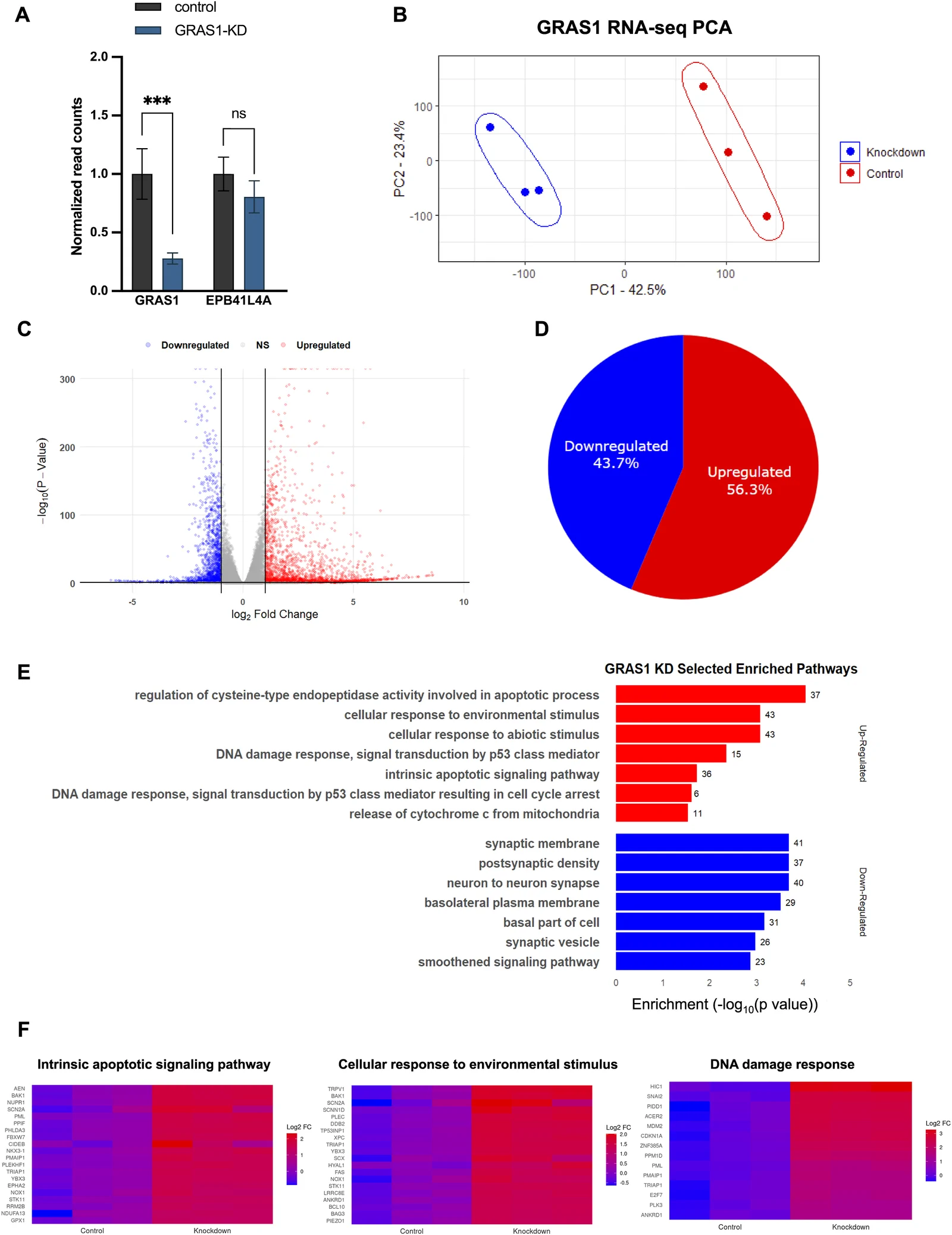
GRAS1 knockdown results in up-regulation of DNA damage response genes. **(A)** RNA expression of GRAS1 and EPB41L4A in A549 cells transfected with control GapmeR compared with GRAS1 GapmeR1, based on read counts in RNA sequencing; N = 3. **(B)** Principal component analysis (PCA) of normalized RNA-seq transcripts per million (TPM) from RNA sequencing of triplicate biological samples of A549 cells transfected with control GapmeR (red) compared with GRAS1 GapmeR1 (blue); N = 3. **(C)** Volcano plot of differentially expressed genes in control GapmeR compared with GRAS1 GapmeR1 knockdown samples in A549 RNA-seq data. Red and blue points mark the genes with significantly increased or decreased expression, respectively. **(D)** Percentage of up-regulated (red) and down-regulated (blue) differentially expressed genes in GRAS1 knockdown cells compared with control cells. **(E)** Gene Ontology enrichment analysis of selected biological processes in up- and down-regulated genes between control GapmeR and GRAS1 GapmeR1–transfected A549 cells. **(F)** Relative expression of the top genes from the intrinsic apoptotic signaling pathway, the cellular response to environmental stimulus, and the DNA damage response after GRAS1 knockdown. Error bars represent SD in all panels; (ns) *P* > 0.05; (***) *P* < 0.001 by two-tailed *t* test.

Because knockdown of GRAS1 led to a dramatic decrease in cell survival, we performed RNA-seq and analyzed the differentially expressed genes after GRAS1 knockdown in A549 lung cancer cells. These datasets were then used to identify altered biological pathways that could contribute to the observed cell death effects. Transcriptome-wide changes in RNA expression in GRAS1 knockdown cells were quantified by Illumina high-throughput next-generation sequencing analysis after 48 h of GapmeR treatment, resulting in 2 × 10^7^–4 × 10^7^ reads per sample (Table S1). The GRAS1 knockdown efficiency by ASO GapmeR was confirmed using read counts from the aligned RNA-seq data in experimental samples compared with control samples. On the other hand, expression level from the neighboring antisense gene *EPB41L4A* of the GRAS1 transcript was not significantly affected by the ASO GapmeR treatment, as quantified using RNA-seq data (Fig 3A).

Table S1. Read statistics for RNA-seq samples in this study.

Principal component analysis (PCA) showed clustering of the three independent biological replicates for each treatment, with a clear separation between knockdown and control samples (Fig 3B). Knockdown of GRAS1 RNA led to significant changes in mRNA expression patterns in A549 lung cancer cells. A total of 3,231 genes were more than twofold differentially expressed with *P*-value ≤0.05 (Fig 3C). The RNA sequencing data are provided on the NCBI Sequence Read Archive BioProject PRJNA979253. In the differentially expressed gene set after GRAS1 knockdown, 56.3% of RNA transcripts were up-regulated and 43.7% of RNA transcripts were down-regulated (Fig 3D). The differentially expressed genes from triplicate biological samples of GRAS1 knockdown versus control GapmeR treatment were used for gene ontology (GO) enrichment analysis, which identified a significant increase in multiple biological pathways related to cell survival and DNA damage response (Fig 3E). Up-regulated transcripts in GRAS1 knockdown cells clustered in pathways including intrinsic apoptotic signaling, cellular response to environmental stimulus, and DNA damage response (Fig 3F). We found that GRAS1 knockdown leads to differential expression of many mRNA transcripts, including those involved in maintenance of genome integrity through the DNA damage response.

### GRAS1 knockdown causes accumulation of multiple DNA damage foci in nuclei

**Figure 4.**
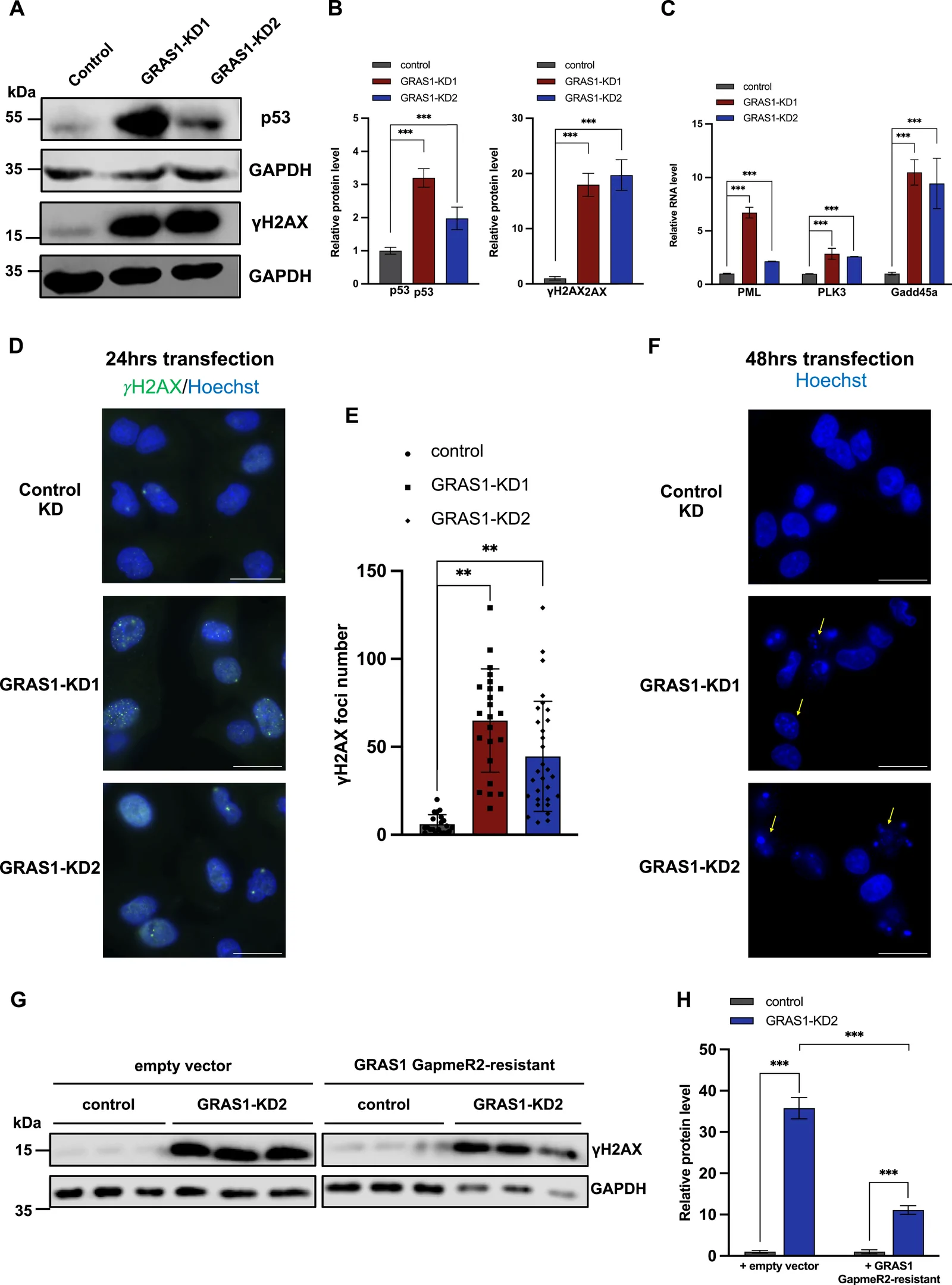
GRAS1 knockdown causes accumulation of multiple DNA damage foci in nuclei. **(A)** Immunoblot of p53 and γH2AX in A549 cells treated with control GapmeR, GRAS1 GapmeR1 or GRAS1 GapmeR2. **(B)** Quantification of immunoblot (A). p53 and γH2AX expression were calculated after normalization to the GAPDH loading control for each sample; N = 4. **(C)** RT-qPCR analysis of PML, PLK3, and Gadd45a expression levels in A549 cells treated with control GapmeR compared with GRAS1 GapmeR1 or GRAS1 GapmeR2, normalized to GAPDH control mRNA; N = 3. **(D)** Representative images of γH2AX foci (green) and Hoechst DNA stain (blue) in A549 cells treated with control GapmeR compared with GRAS1 GapmeR1 or GRAS1 GapmeR2 after 24 h of transfection. The scale bar represents 25 μm. **(E)** Quantification of (D) showing γH2AX foci per cell from three independent immunofluorescence experiments. **(F)** Representative images of Hoechst DNA stain (blue) in A549 cells treated with control GapmeR compared with GRAS1 GapmeR1 or GRAS1 GapmeR2 after 48 h of transfection. Yellow arrows indicate regions of nuclear fragmentation. The scale bar represents 25 μm. **(G)** Immunoblot of γH2AX in A549 cells co-transfected with empty vector or GRAS1 GapmeR–resistant vector, and with control GapmeR or GRAS1 GapmeR2. **(H)** Quantification of immunoblot (G). γH2AX expression was calculated after normalization to the GAPDH loading control for each sample; N = 3. Error bars represent SD in all panels; (**) *P* ≤ 0.01; (***) *P* < 0.001 by two-tailed *t* test.

Based on the gene expression alterations in pathways related to apoptotic signaling, cellular response, and DNA damage response, we next investigated whether cellular phenotypes associated with DNA damage occurred in GRAS1 knockdown cells. The protein p53 is a key regulator of cell viability and cell cycle progression in response to DNA damage and other types of cell stress (Kastan et al, 1995). Knockdown of GRAS1 significantly increased p53 protein levels after treatment with ASO GapmeRs (Fig 4A and B). The mRNA expression levels of p53 downstream gene targets including PML, PLK3, and Gadd45a were also significantly increased (Fig 4C). Consistent with the results of our RNA-seq analysis, we observed that down-regulation of the GRAS1 ncRNA transcript activates p53 signaling in A549 cells.

The p53 signaling pathway can be activated by DNA damage (Lakin & Jackson, 1999). Based on the observation of a strong up-regulation of non-homologous end joining DNA damage response genes by RNA-seq, we next evaluated whether knockdown of GRAS1 increases double-strand breaks and DNA damage in lung cancer cells. The histone variant H2AX is rapidly phosphorylated at serine 139 (γH2AX) in an early cellular response to DNA double-strand breaks (Mah et al, 2010). Histone H2AX phosphorylation was measured by immunoblotting in GRAS1 knockdown A549 cells as an indicator of DNA damage. Knockdown of GRAS1 significantly increased the overall levels of γH2AX compared with control cells (Fig 4A and B). We then enumerated γH2AX foci in individual cells using immunofluorescence microscopy. Discrete γH2AX foci in cell nuclei correlate with the locations of DNA double-strand breaks (Mah et al, 2010). Consistent with the results of γH2AX immunoblotting, we found that knockdown of GRAS1 significantly increased the number of γH2AX foci. Many individual cells contained more than 100 γH2AX foci only 24 h after GRAS1 knockdown (Fig 4D and E). In comparison, control cells showed fewer than 10 γH2AX foci per cell. These results indicate that down-regulation of GRAS1 leads to a significant increase in DNA damage and double-strand breaks in A549 cells.

Chromosome condensation and DNA fragmentation is a typical characteristic of cell death after extensive DNA damage (Toné et al, 2007). Knockdown of GRAS1 severely affected the nuclear morphology in A549 cells after 48 h of GapmeR treatment, with the percentage of cells with irregular nuclei measured at 3.7% in control GapmeR-treated cells, 40.2% in GRAS1 GapmeR1–treated cells, and 39.9% in GRAS1 GapmeR2–treated cells, respectively (Fig 4F). DNA damage induced by GRAS1 knockdown was partially rescued by complementation with GapmeR-resistant GRAS1 overexpression, confirming the essential role of GRAS1 in protecting cells against DNA damage (Fig 4G and H).

**Figure S4.**
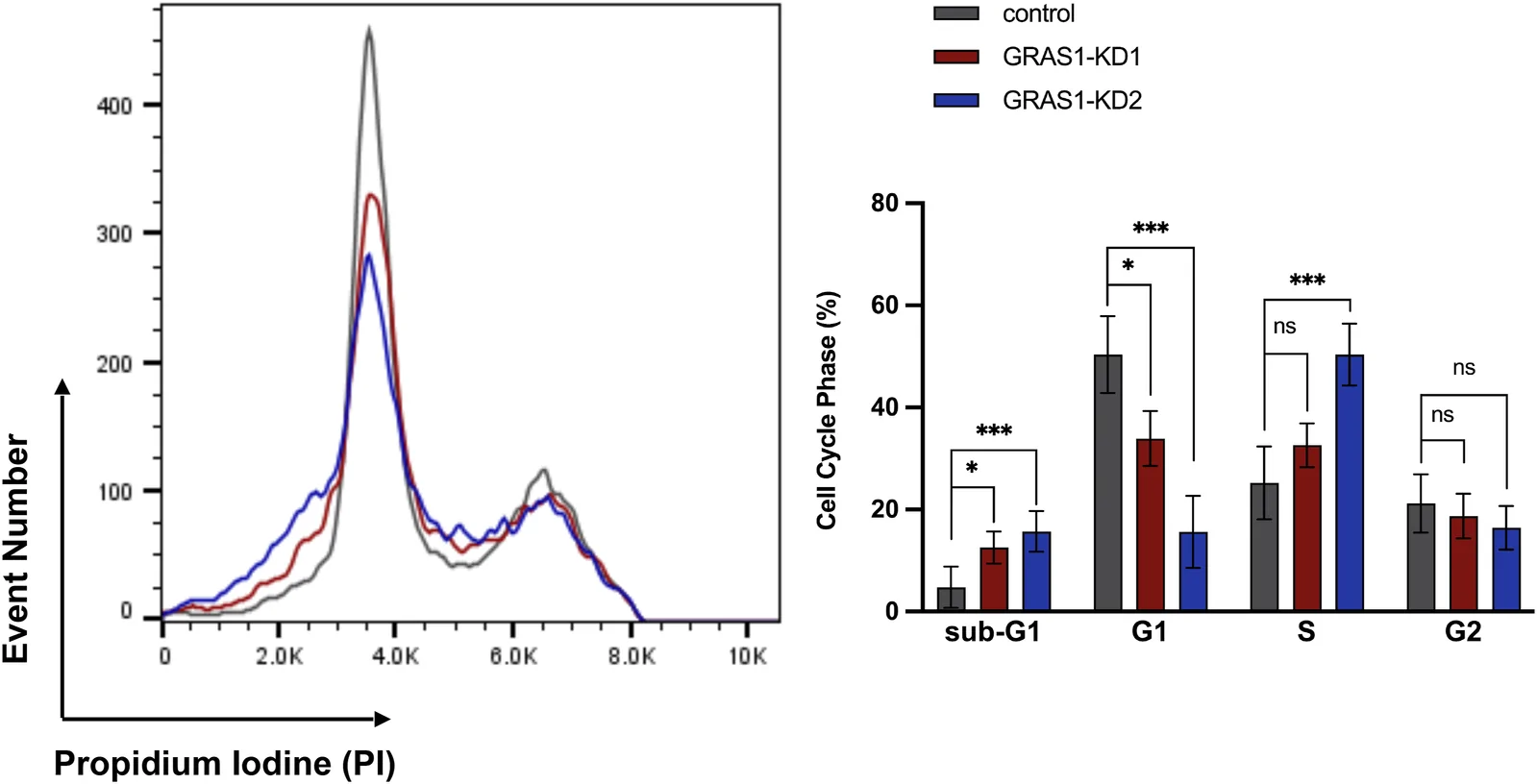
GRAS1 knockdown affects cell cycle distribution. Representative images of cell cycle analysis by propidium iodide staining using flow cytometry of A549 cells treated with control GapmeR compared with GRAS1 GapmeR1 or GRAS1 GapmeR2 (left). Quantification of the proportion of cells in each cell cycle phase was performed from three independent experiments (right). Error bars represent SD in all panels; (ns) *P* > 0.05; (*) *P* ≤ 0.05; (***) *P* < 0.001 by two-tailed *t* test.

To evaluate the impacts of the GRAS1 knockdown on cell cycle progression, we performed flow cytometry with propidium iodide staining, and data were quantified from three independent experiments. We found that GRAS1 knockdown affected A549 cell cycle distribution, with a significant increase in the cell populations in S-phase and sub-G1 phase (Fig S4). In contrast, the proportion of cells in the G1 phase decreased significantly after GRAS1 knockdown. Overall, we conclude that the down-regulation of GRAS1 induces extensive cell cycle dysregulation and DNA damage, with multiple double-strand breaks in A549 cells leading to cell death.

### GRAS1 directly binds and stabilizes NKAP

**Figure 5.**
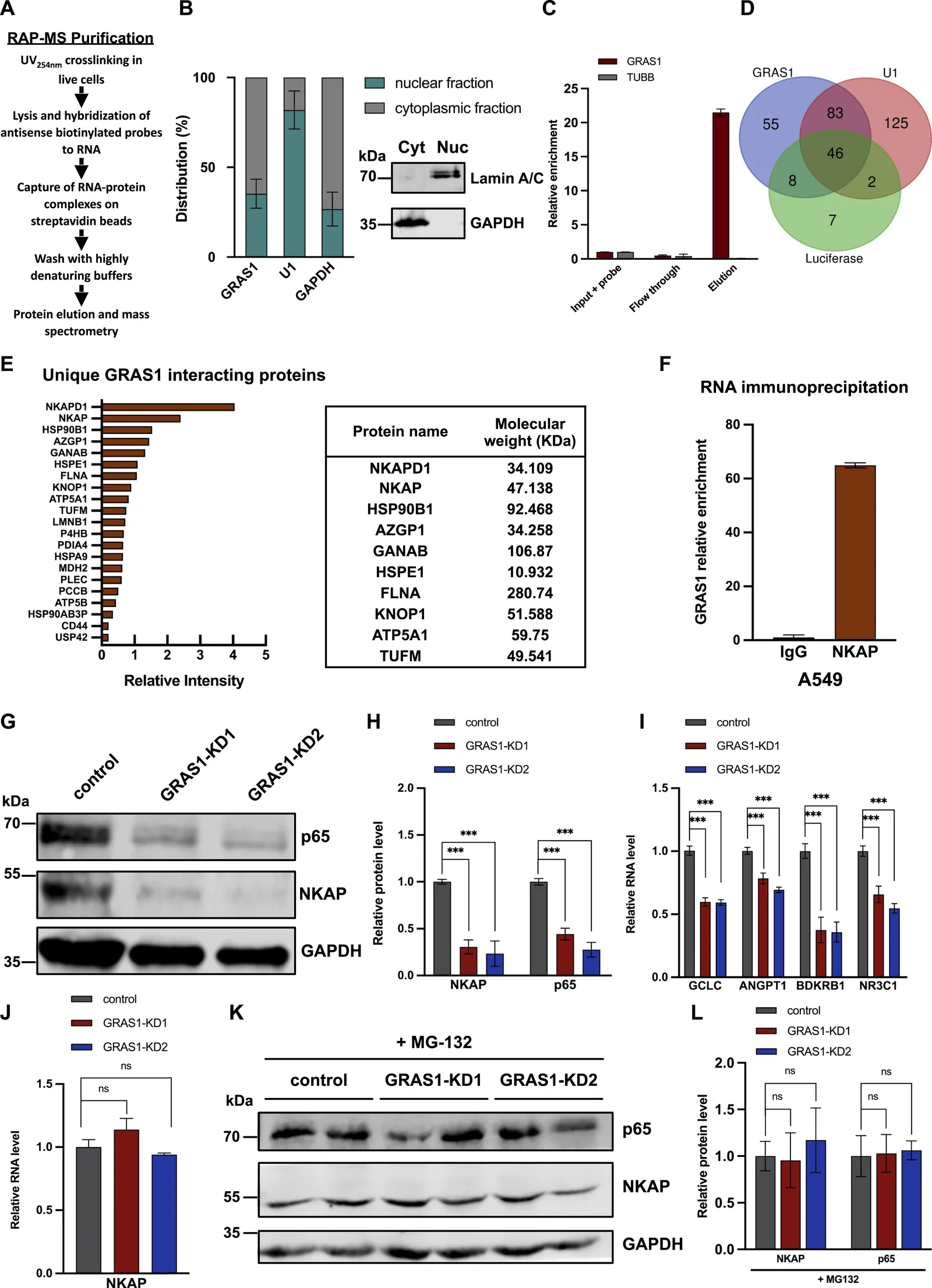
GRAS1 directly binds and stabilizes NKAP. **(A)** RNA antisense purification–mass spectrometry experimental outline. **(B)** RT-qPCR analysis of GRAS1, U1 snRNA, and GAPDH mRNA in nuclear and cytoplasmic fractionations of A549 cells (left panel). Immunoblot of Lamin A/C as a marker for nuclear fraction, and GAPDH as a marker for cytoplasmic fraction (right panel); N = 3. **(C)** Validation of GRAS1 RNA capture and recovery by biotinylated antisense oligonucleotide probes against GRAS1. RT-qPCR analysis of GRAS1 RNA and TUBB control mRNA in three fractions, including input with probes added, unbound sample flowthrough, and elution; N = 3. **(D)** Total proteins identified in RAP-MS of GRAS1 RNA, U1 snRNA positive control, and luciferase mRNA negative control elution samples. **(E)** GRAS1 interactome proteins identified by RAP-MS, with at least two unique peptides, after excluding proteins that were also found in U1 and luciferase control captures (left panel). The top 10 GRAS1-interacting proteins and their molecular weights (right panel). **(F)** GRAS1 RNA enrichment measured by RT-qPCR after immunoprecipitation of NKAP in A549 cells, compared with immunoprecipitation with IgG control antibody; N = 3. **(G)** Immunoblot of NKAP and p65 in A549 cells treated with control GapmeR compared with GRAS1 GapmeR1 or GRAS1 GapmeR2. **(H)** Quantification of immunoblot (G). NKAP and p65 expression were calculated after normalization to the GAPDH loading control for each sample; N = 3. **(I)** RT-qPCR analysis of GCLC, ANGPT1, BDKRB1, and NR3C1 expression levels in A549 cells treated with control GapmeR compared with GRAS1 GapmeR1 or GRAS1 GapmeR2, normalized to GAPDH control mRNA; N = 3. **(J)** RT-qPCR analysis of NKAP mRNA expression in A549 cells treated with control GapmeR compared with GRAS1 GapmeR1 or GRAS1 GapmeR2, normalized to GAPDH control mRNA; N = 3. **(K)** Immunoblot of NKAP and p65 in A549 cells treated with control GapmeR compared with GRAS1 GapmeR1 or GRAS1 GapmeR2, with 5 μM MG-132 treatment. **(L)** Quantification of immunoblot (K). NKAP and p65 expression were calculated after normalization to GAPDH loading control for each sample; N = 6. Error bars represent SD in all panels; (ns) *P* > 0.05; (***) *P* < 0.001 by two-tailed *t* test.

To investigate the biological mechanism by which GRAS1 regulates cell growth, we identified direct protein interaction partners of the endogenous GRAS1 RNA transcript in A549 cells using RNA antisense purification with mass spectrometry (RAP-MS) (Fig 5A). RAP-MS identifies only direct and specific RNA–protein interaction partners by covalently cross-linking “zero-distance” binding interactions between nucleobases and aromatic amino acids with UV_254nm_ light. The use of a highly denaturing wash buffer containing 4 M urea and 500 mM lithium chloride also eliminates indirect and nonspecific protein interactions from the capture sample (McHugh et al., 2014, 2015; Trang et al, 2023). This method identifies only direct RNA–protein interactions. We examined the localization of GRAS1 by subcellular fractionation. GRAS1 was detected in both A549 nuclear and cytoplasmic fractions by RT-qPCR. Control GAPDH mRNA was localized in the nucleus and cytoplasm and U1 snRNA was localized predominantly to the nucleus, in line with the known cellular functions of these RNA transcripts (Fig 5B). Based on this dual localization, we used whole cell lysate for RAP-MS assay to identify GRAS1 interactors in both the nucleus and the cytoplasm. Efficient capture and recovery of endogenous GRAS1 RNA with biotinylated DNA antisense probes was validated in biological triplicate samples (Fig 5C). A single large-scale RAP-MS capture experiment was then performed to purify endogenous RNA–protein complexes from UV_254nm_ cross-linked cells and identify potential direct binding partners of the GRAS1 ncRNA. The GRAS1 ncRNA capture from served as the experimental sample, whereas a U1 snRNA capture was used as a positive control and a luciferase mRNA capture was used as a negative control for the RAP-MS experiment (Fig 5D). Analysis of LC-MS/MS for unique peptides using MaxQuant identified 63 background proteins in the luciferase control capture, 256 proteins in the U1 control capture, and 192 proteins in the GRAS1 capture sample. After subtracting background proteins that were also identified in the luciferase negative control sample, 138 proteins were specific for the GRAS1 capture sample and 208 proteins were specific for the U1 capture sample. To eliminate commonly identified RNA-binding proteins and splicing factors, we next removed any proteins which were also found in the U1 snRNA capture from the GRAS1 protein list. Finally, the GRAS1 protein list was filtered to include only proteins identified with at least two unique peptides, leaving 21 proteins identified as direct interaction partners of GRAS1 ncRNA by RAP-MS (Fig 5E). Mass spectrometry datasets as well as MaxQuant peptide search results and parameter files are available at ProteomeXchange with identifier PXD042699.

NKAP and the protein NKAP domain containing 1 (NKAPD1) were identified in our MS screen as the top interaction partners of GRAS1 ncRNA. Consistent with our finding of direct binding to GRAS1 RNA, NKAP was previously characterized as an RNA-binding protein (Burgute et al, 2014). To validate the interaction between GRAS1 and NKAP with an orthogonal approach, we performed RNA immunoprecipitation (RIP) in non-crosslinked A549 cell lysates, by capturing NKAP and testing for the presence of GRAS1 by qRT-PCR. We observed more than a 50-fold increase in GRAS1 RNA capture in NKAP-IP sample elution compared with the control IgG sample elution (Fig 5F). The molecular function of NKAPD has not been extensively characterized. Therefore, we focused on the functions of the NKAP protein to investigate whether the direct interaction between GRAS1 and NKAP contributes to cell survival and protection against DNA damage.

Because GRAS1 directly interacts with NKAP, we investigated the effect of GRAS1 knockdown on NKAP mRNA and protein levels. Knockdown of GRAS1 led to a significant decrease in NKAP protein levels (Fig 5G–I). Notably, loss of GRAS1 did not significantly affect NKAP mRNA levels (Fig 5J). Therefore, we hypothesized that GRAS1 RNA affects NKAP through a posttranslational regulatory mechanism. Treatment of cells with the proteasome and calpain inhibitor MG-132 reduced the effect of GRAS1 knockdown on the protein level of NKAP (Fig 5K and L). The combined data from RAP-MS and RNA immunoprecipitation experiments indicated that GRAS1 ncRNA binds directly to NKAP in A549 cells, and that this interaction affects NKAP stability through a posttranslational regulatory mechanism.

NKAP was initially identified as an activator of the NF-κB pathway. To further investigate the effects of GRAS1 perturbation on NF-κB activation and proliferative signaling, we also measured the effect of GRAS1 knockdown and subsequent loss of NKAP on this pathway in GapmeR-treated cells. The level of the NF-κB p65/RelA subunit protein was significantly decreased after the loss of GRAS1 and NKAP (Fig 5H and I), and MG-132 treatment was similarly protective for p65/RelA levels after GRAS1 knockdown (Fig 5K and L). Consistent with the fact that NKAP was identified as an activator of this pathway, knockdown of GRAS1 also resulted in decreased mRNA expression levels of the NF-κB target genes GCLC, ANGPT1, BDKRB1, and NR3C1. GAPDH control levels remained unaffected in each experiment. These additional data support the role of GRAS1 transcript and NKAP in promoting cell growth through the NF-κB pathway.

### GRAS1 stabilizes NKAP to protect against cell death and DNA damage

**Figure 6.**
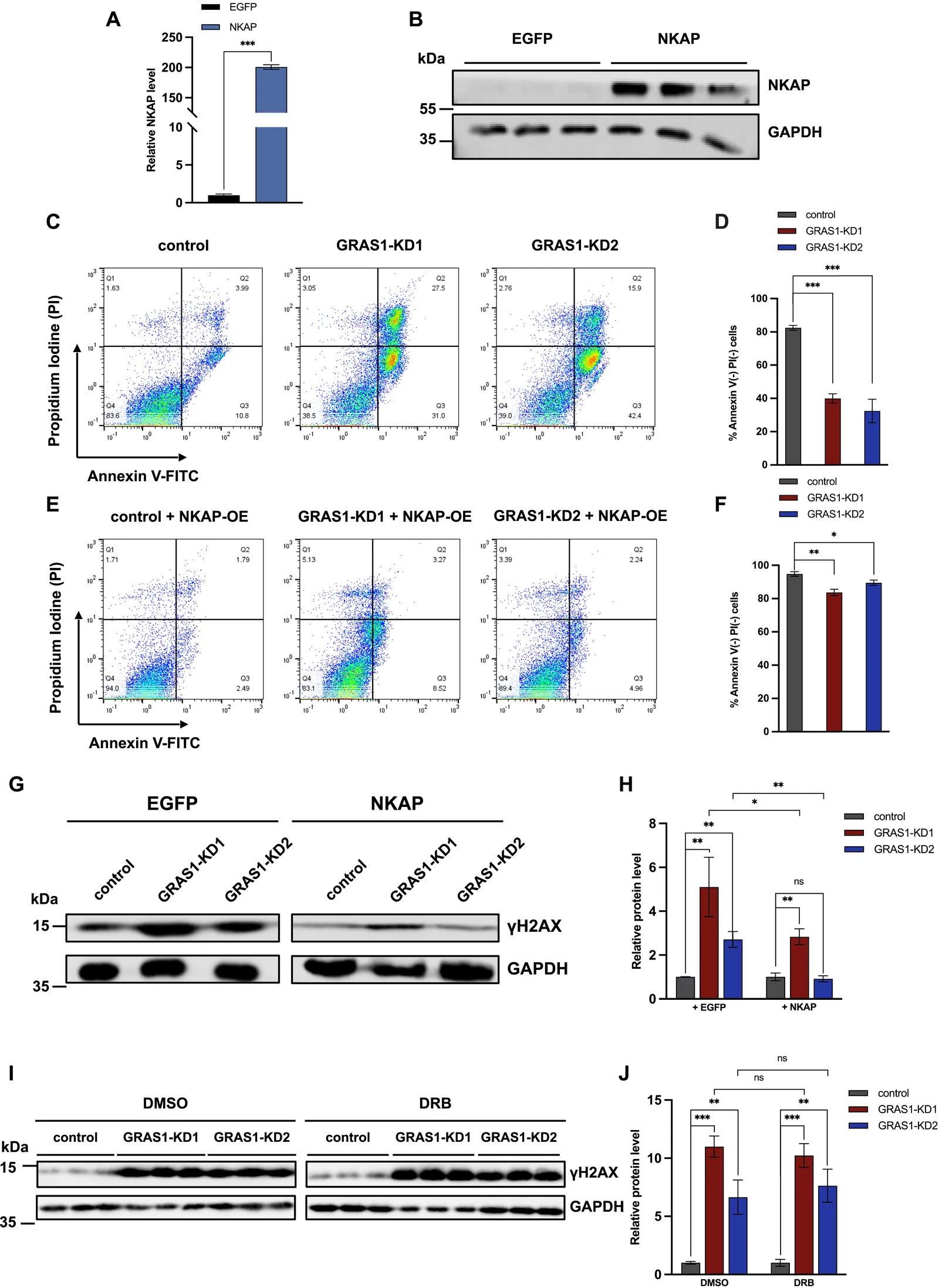
GRAS1 stabilizes NKAP to protect cells against cell death and DNA damage. **(A)** RT-qPCR analysis of NKAP mRNA expression in A549 cells treated with EGFP control vector compared with NKAP vector, normalized to GAPDH control mRNA; N = 3. **(B)** Immunoblot of NKAP in A549 cells treated with EGFP control vector compared with NKAP vector; N = 3. **(C)** Representative results for flow cytometry of A549 cells treated with control GapmeR, GRAS1 GapmeR1, or GRAS1 GapmeR2. **(D)** Quantification of (C) showing the percentage of Annexin V–negative/PI-negative A549 cells treated with control GapmeR compared with GRAS1 GapmeR1 or GRAS1 GapmeR2; N = 3. **(E)** Representative results for flow cytometry of A549 cells co-transfected with NKAP vector and with control GapmeR, GRAS1 GapmeR1, or GRAS1 GapmeR2. **(F)** Quantification of (E) showing the percentage of Annexin V–negative/PI-negative A549 cells co-transfected with NKAP vector and with control GapmeR, GRAS1 GapmeR1, or GRAS1 GapmeR2; N = 3. **(G)** Immunoblot of γH2AX in A549 cells co-transfected with EGFP control vector or NKAP vector, and with control GapmeR, GRAS1 GapmeR1, or GRAS1 GapmeR2. **(H)**. Quantification of immunoblot (G). γH2AX expression was calculated after normalization to the GAPDH loading control for each sample; N = 3. **(I)** Immunoblot of γH2AX in A549 cells treated with control GapmeR, GRAS1 GapmeR1, or GRAS1 GapmeR2 for 46 h, followed by DMSO or 100 μM DRB treatment for 2 h. **(J)** Quantification of immunoblot (I). γH2AX expression was calculated after normalization to the GAPDH loading control for each sample; N = 3. Error bars represent SD in all panels; (ns) *P* > 0.05; (*) *P* ≤ 0.05; (**) *P* ≤ 0.01; (***) *P* < 0.001 by two-tailed *t* test.

To determine whether DNA damage resulting from NKAP depletion is a main contributor to cell death after GRAS1 knockdown, we performed rescue experiments with NKAP ectopic expression in GRAS1 knockdown cells. We achieved high overexpression efficiency using the NKAP plasmid compared with the EGFP control plasmid, as validated by RT-qPCR for NKAP mRNA level (Fig 6A) and immunoblotting for NKAP protein level (Fig 6B). As observed previously, treatment with GRAS1 GapmeR significantly decreased cell survival, compared with treatment with control GapmeR (Fig 6C and D). By flow cytometry, the apoptotic cell population marked by Annexin V staining was significantly increased in GRAS1 knockdown cells. We observed that NKAP expression protected cells from apoptotic death and abrogated the effect of GRAS1 knockdown (Fig 6E and F). Finally, we tested if expression of NKAP could reverse the effect of GRAS1 depletion on DNA damage. Overexpression of NKAP slightly decreased the levels of γH2AX in GRAS1 knockdown cells treated with ASO GapmeR 1 and significantly decreased the γH2AX levels in GRAS1 knockdown cells treated with ASO GapmeR 2 (Fig 6G and H). These results indicate that complementation with NKAP overexpression protects against the cellular effects of GRAS1 down-regulation.

A recent study has proposed that NKAP is involved in preventing the accumulation of R-loops by interacting with HDAC3. The formation of R-loops occurs during active transcription and results in DNA damage and genome instability (Zhang et al, 2023). Because GRAS1 binds and protects NKAP from degradation, we asked whether GRAS1 contributes to the functionality of NKAP/HDAC3 complex in preventing R-loops accumulation and DNA damage. To test this, we treated cells with 5,6-dichloro-1-beta-D-ribofuranosylbenzimidazole (DRB), an inhibitor for RNA polymerase II, to block transcription and R-loops formation, and see whether the DNA damage induction caused by GRAS1 knockdown could be rescued. As previously observed, γH2AX protein levels dramatically increased in GRAS1 depleted cells, but the DRB treatment did not alleviate the DNA damage caused by GRAS1 knockdown (Fig 6I and J). This suggests that cellular effects of GRAS1 knockdown cannot be attributed to R-loop formation. Altogether, we found that NKAP stabilization by GRAS1 is important for protecting cells against cell death and DNA damage using a mechanism different from R-loop associated pathway.

### GRAS1 function is conserved in HCT116 colorectal cancer cells

**Figure 7.**
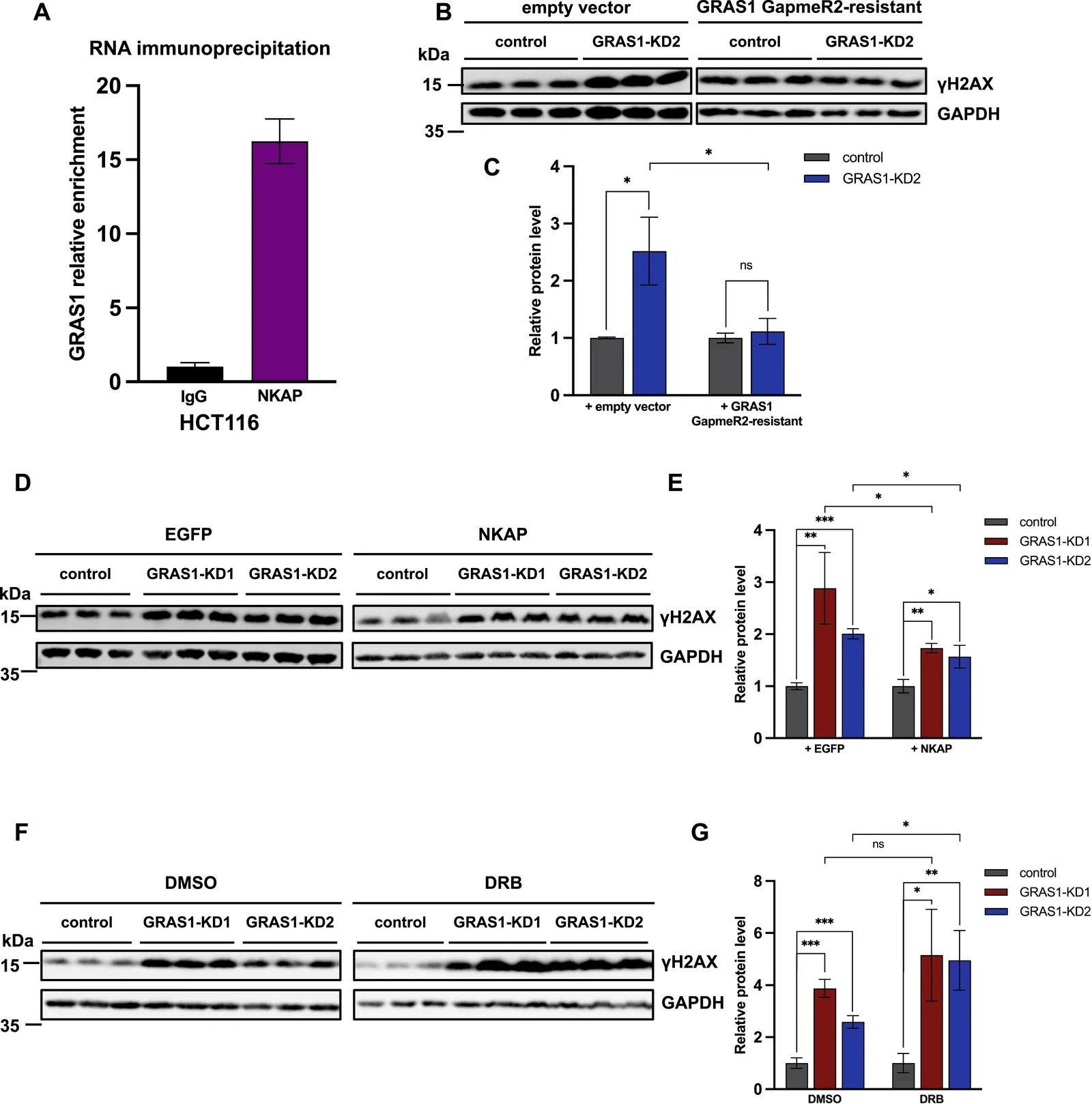
GRAS1 function is conserved in HCT116 colorectal cancer cells. **(A)** GRAS1 RNA enrichment measured by RT-qPCR after immunoprecipitation of NKAP in HCT116 cells, compared with immunoprecipitation with IgG control antibody; N = 3. **(B)** Immunoblot of γH2AX in HCT116 cells co-transfected with empty vector or GRAS1 GapmeR–resistant vector, and with control GapmeR or GRAS1 GapmeR2. **(C)** Quantification of immunoblot in panel (B); γH2AX expression was calculated after normalization to the GAPDH loading control for each sample; N = 3. **(D)** Immunoblot of γH2AX in HCT116 cells co-transfected with EGFP vector or NKAP vector, and with control GapmeR, GRAS1 GapmeR1, or GRAS1 GapmeR2. **(E)** Quantification of immunoblot in panel (D). γH2AX expression was calculated after normalization to the GAPDH loading control for each sample; N = 3. **(F)** Immunoblot of γH2AX in HCT116 cells treated with control GapmeR, GRAS1 GapmeR1, or GRAS1 GapmeR2 for 46 h, followed by DMSO or 100 μM DRB treatment for 2 h. **(G)** Quantification of immunoblot in panel (F). γH2AX expression was calculated after normalization to the GAPDH loading control for each sample; N = 3. Error bars represent SD in all panels; (ns) *P* > 0.05; (*) *P* ≤ 0.05; (**) *P* ≤ 0.01; (***) *P* < 0.001 by two-tailed *t* test.

The cell proliferation assay indicated that GRAS1 is essential for cell growth and survival in multiple cancer cell lines (Fig 2A). We further investigated whether GRAS1 regulates similar cellular pathways in HCT116 colorectal cancer cells as in A549 lung cancer cells. Both the A549 and HCT116 cell lines are obtained from tumors of epithelial origin and express the wild-type p53 protein. We validated the interaction between GRAS1 and NKAP in HCT116 cells by RNA immunoprecipitation assay and observed significant increase in GRAS1 captured by NKAP antibody compared with control IgG antibody (Fig 7A). In addition, we found that GRAS1 knockdown in HCT116 cells also resulted in a significant increase in γH2AX protein level, demonstrating an induction of DNA damage leading to cell death, similar to the effect observed in A549 cells. This phenotype was fully complemented by the overexpression of the GapmeR-resistant GRAS1 transcript (Fig 7B and C). Overexpression of NKAP partially protected cells against DNA damage induced by GRAS1 depletion (Fig 7D and E), indicating that GRAS1 and NKAP interaction and function are similar in HCT116 cells and A549 cells. Furthermore, treatment with DRB did not rescue the DNA damage induced by GRAS1 depletion in HCT116 cells, similar to the observation for A549 cells (Fig 7F and G). Overall, we confirmed that GRAS1 function is conserved in HCT116 colorectal cancer cells, and that the interaction between GRAS1 and NKAP is important for DNA damage protection in more than one cell type.

## Discussion

Cellular functions of many of the noncoding RNAs produced by the human genome are still uncharacterized. CRISPR/Cas9 screens have identified growth effects for noncoding RNA transcripts in several cell types, but the interaction pathways and mechanisms of RNA function remain generally unknown. Here, we identified a new mechanism for GRAS1 noncoding RNA in promoting cell survival by directly interacting with NKAP to protect against DNA damage.

The *EPB41L4A-AS1* gene was identified as a growth regulator locus in several earlier studies. At least three biomolecules are produced from this multifunctional gene locus: the small TIGA1 protein, the SNORA13 transcript, and the GRAS1 long noncoding RNA which was examined in further detail here. Our results suggest that the expression of GRAS1 noncoding RNA in actively growing cells promotes proliferation and growth. Conversely, expression of TIGA1 and SNORA13 are most relevant in quiescent and senescent cells, and each shows the opposite effect from GRAS1 by inhibiting proliferation of actively growing cells. Whereas the specific biological function of TIGA1 remains unclear, the protein is predicted to act as a mitochondrially localized ion transporter. The production of TIGA1 protein has been shown to be strongly up-regulated upon growth arrest, after at least 3 d of serum and amino acid starvation (Yabuta et al, 2006). Further study of the transcriptional regulation of each of these individual transcripts would be critical to unravel the full functionality of the *EPB41L4A-AS1* locus.

Here, we focused on identifying some cellular pathways by which GRAS1 noncoding RNA could promote growth in lung and colon cancer cell lines. RNA sequencing analysis showed a strong up-regulation in DNA damage responses after loss of GRAS1 in A549 cells, indicating that the expression of GRAS1 in normal cell growth is protective. A common role for GRAS1 in the protection of genome stability was revealed in both the non–small-cell lung cancer cell line A549 and the colorectal cancer cell line HCT116. A previous report focused on the role of gene products from the *EPB41L4A-AS1 *locus in regulating glycolysis (Liao et al, 2019), whereas our study additionally revealed a strong DNA damage response due to GRAS1 depletion in A549 and HCT116 cell lines.

It is unclear why DNA damage and cell death occur in A549 and HCT116 cell lines but are not observed in HEK293 cells in response to loss of GRAS1. Cell type differences in gene expression or p53 function may affect GRAS1 sensitivity. In our study and others, perturbation of the *EPB41L4A-AS1* locus was observed to affect p53 activation. Both A549 and HCT116 cells are of epithelial origin and express wild-type p53. HEK293 cells have been proposed to have high levels of constitutively active p53 (Chee et al, 2013). Mammalian cells generally maintain a low level of p53, because of continual degradation and rapid turnover. Cellular p53 accumulation can be caused by multiple stressors, including DNA damage (Lakin & Jackson, 1999). GRAS1 knockdown induced p53 activation, in line with our RNA-seq data and microscopic observations of extensive DNA damage and nuclear fragmentation after GRAS1 depletion.

Using an unbiased proteomics assay for direct binding partners, we found that GRAS1 binds directly to NKAP, a protein which promotes accurate chromosome segregation and cell survival in HeLa cells. Inhibition of proteasomal degradation prevented NKAP degradation upon GRAS1 knockdown, so we hypothesize that GRAS1 could potentially stabilize NKAP either through directly interacting with the protein or by indirectly affecting other factors involved in NKAP degradation. NKAP function has previously been shown to be required for highly proliferative cell survival and for accurate neuronal development in children. Dysregulation of NKAP causes chromosome segregation defects and genome instability in HeLa cells (Li et al, 2016) and loss of NKAP increases the number of DNA damage foci in U2-OS cells (Zhang et al, 2023). The decrease in cell survival after GRAS1 knockdown was rescued by ectopic expression of NKAP or GRAS1, indicating that GRAS1–NKAP interaction is directly related to survival in A549 lung cancer cells and HCT116 colorectal carcinoma cells. The mechanism by which GRAS1 noncoding RNA binding could stabilize NKAP is still uncharacterized, although a few potential mechanisms may be proposed. GRAS1 binding could physically occlude access to NKAP by enzymes responsible for sumoylation, degradation, or other modifications on NKAP that regulate function. Alternatively, GRAS1 binding could affect the protein structure in the predicted disordered regions of NKAP to prevent aggregation or degradation of NKAP. Because NKAP has recently been identified as an essential protein involved in MRXSHD developmental disorder, we believe that a more detailed investigation of the mechanisms and regulation of NKAP turnover and stabilization would be warranted in future studies. In A549 cells, we also found that the normal cell cycle was disrupted with significant accumulation of cells in S-phase and sub-G1 phase after GRAS1 knockdown, suggesting that GRAS1 expression and protection from DNA damage may be required for normal S-phase progression. NKAP expression protects against mitotic collapse in HeLa cells, so it is possible that the stabilization of NKAP by GRAS1 similarly protects against mitotic collapse in the cell lines studied here. However, future work would be needed to uncover the specific details of how GRAS1 and NKAP can promote normal cell growth and protect genome integrity.

We conclude that the GRAS1–NKAP interaction protects against DNA damage and promotes growth in multiple human cell lines. Regulation of RNA and protein expression levels is an important mechanism of cellular control during cancer development and metastasis. The multifunctional *EPB41L4A-AS1* gene locus produces at least three biologically active molecules—TIGA1 protein, SNORA13 snoRNA, and GRAS1 noncoding RNA. TIGA1 and SNORA13 have previously been proposed to play a role in cellular quiescence and senescence, whereas our study shows that the noncoding RNA GRAS1 interacts with NKAP to promote growth in lung and colon cancer cell lines. Multifunctional human gene loci producing both RNA and protein biomolecules may enable flexible context-dependent activity in specific cell states. Additional studies will be required to elucidate the mechanism of GRAS1 regulation of NKAP stability, and to evaluate the contributions of GRAS1 RNA and other products of the *EPB41L4A-AS1* locus to tumor development and metastasis.

## Materials and Methods

### Cell lines and culture conditions

Human lung adenocarcinoma A549 cell line (ATCC No. CCL-185), human colorectal carcinoma HCT116 cell line (ATCC No. CCL-247), and human kidney embryonic HEK293 cell line (ATCC No. CRL-1573) were purchased from ATCC. A549 and HEK293 cells were cultured in Eagle’s Minimal Essential Medium (EMEM) (Quality Biological); HCT116 cells were cultured in McCoy’s 5A Modified Medium (Gibco, Life Technologies). All media were supplemented with 10% fetal bovine serum (Gibco, Life Technologies) and 1% additional L-Glutamine (Corning). All cells were grown in a cell culture incubator at 37˚C with 5% CO_2_. Cells were tested monthly for mycoplasma.

### Plasmid generation and transfection

Gene inserts were generated by PCR from A549 or K562 complementary DNA (cDNA) using locus-specific primers. Entry vectors of the Gateway cloning system were created with the pENTR/D-TOPO Cloning Kit (2402649; Invitrogen). Once the entry vectors were generated, the gene inserts were transferred to pcDNA3.2 destination vectors using the Gateway LR Clonase Enzyme Mix (11791011; Thermo Fisher Scientific). For plasmid transfections, cells were seeded with 2 × 10^5^ cells/well in 12-well plates overnight before each treatment. Cells in each well were transfected with 1 μg of plasmid using 3.5 μl of Lipofectamine 2000 (Thermo Fisher Scientific) in Opti-MEM I reduced serum medium (31985062; Life Technologies) following the manufacturer’s suggested protocol.

### ASO GapmeR knockdown of GRAS1

For GRAS1 RNA knockdown, cells in each well were transfected with 50 μM of each ASO locked nucleic acid GapmeR1 or GapmeR2 (Qiagen, custom design) with Negative Control GapmeR A (30300019-2; Qiagen) using 3.5 μl of Lipofectamine 2000 in Opti-MEM I reduced serum medium following the manufacturer’s suggested protocol. Samples were incubated for 24 or 48 h before collection, as indicated in the text. GapmeR target sequences were: GRAS1 GapmeR1 sequence (AAATGATCCAGGCAAC) and GRAS1 GapmeR2 sequence (ACG​CTG​CTT​TTA​AGT​T).

### Nucleic acid isolation and qRT-PCR

Total RNA from all cell treatments was isolated using QIAshredder and RNeasy Mini kit (Qiagen) then further purified or concentrated as needed with RNA Clean & Concentrator kit (Zymo Research). For cell fractionation experiments, total RNA from each cell fraction was isolated using Qiagen RNeasy columns. DNA isolations were performed using plasmid Miniprep kit (Qiagen). All isolations were performed according to the manufacturer’s protocol. 1.5 μg of purified RNA was used for each reverse transcription reaction with 1 μl of Superscript III Reverse Transcriptase (18080085; Invitrogen). Quantitative real-time PCR (qRT-PCR) was performed using ROX Reference Dye (Invitrogen) and SYBR Green Dye (Life Technologies), with fold expression calculated using the 2^−ΔΔCt^ method. Samples were repeated in biological triplicate. Data were normalized to IgG for immunoprecipitation experiments, normalized to GAPDH or tubulin control genes for qRT-PCR experiments, and normalized to respective RNA amounts for cellular fractionation products. Primers used for qRT-PCR experiments and cloning of transcripts are provided in Table S2.

Table S2. Oligonucleotide primer sequences for qRT-PCR and cloning.

### Immunoblotting

After transfection, cells were harvested with trypsin and washed with 1X PBS. Cells were then mixed with cell lysate buffer, protein inhibitor cocktail (539134; Millipore Sigma), sodium fluoride, and incubated at 95°C for 10 min before SDS–PAGE separation. Cell lysates were loaded into a 10-well 10% Bis–Tris SDS-polyacrylamide gel along with PageRuler Plus Protein ladder (26619; Thermo Fisher Scientific). Proteins were separated by electrophoresis at 130 V for 90 min and subsequently transferred to 0.2 micron pore size nitrocellulose membrane. Membranes were blocked with the LI-COR Biosciences Intercept Blocking buffer (VWR International) for one h at room temperature and incubated with primary antibodies overnight at 4°C on a rocker. The following primary antibodies were used: NKAP polyclonal antibody (ab229096; Abcam, 1:1,000 dilution), phospho-histone (γ) H2A.X Ser139 20E3 (60566SF; Cell Signaling Technology, 1:1,000 dilution), p53 (10442-1-AP; Proteintech, 1:1,000 dilution), GAPDH (60004-1-Ig; Proteintech, 1:3,000 dilution), Lamin A/C (10298-1-AP; Proteintech, 1:1,000 dilution), NF-κB p65 (10745-1-AP; Proteintech, 1:1,000 dilution), and TIGA1 (MBS9610109; MyBioSource, 1:1,000 dilution). All antibodies were diluted in 1X TBST. After incubation, membranes were washed four times for 5 min with 1X TBST buffer (1X TBS with 0.1% Tween 20). Nitrocellulose membrane was then incubated with goat Anti-mouse (102971-154; VWR International) and goat Anti-rabbit (103011-498; VWR International) IRDye-conjugated secondary antibodies for one h under dark conditions on a rocker. After four 5-min washes, the membrane was imaged with an Odyssey Fc device (LI-COR Bioscience) and analyzed with Empiria Studio software (LI-COR Bioscience).

### Cellular fractionation

For separation of cytoplasmic and nuclear components, the protocol was performed essentially as previously reported (Gagnon et al, 1999) Briefly, 1 × 10^7^ A549 cells were collected by trypsinization and resuspended in 1 ml hypotonic lysis buffer (10 mM Tris–HCl [pH 7.5], 10 mM NaCl, 3 mM MgCl_2_, 0.3% NP-40 supplemented with Complete protease inhibitor cocktail (Roche) and 10% glycerol). After 10 min of incubation on ice and a brief vortex of the sample, 60 μl input control for immunoblot was reserved. The remaining lysate was centrifuged for 8 min, 800*g* at 4°C. The supernatant was separated and saved as the cytoplasmic fraction, and 5 M NaCl was added to create a final concentration of 140 mM NaCl in the sample. The remaining pellet was washed four times by resuspending in hypotonic lysis buffer and centrifuged for 2 min at 300*g*. The supernatants were saved as individual washes. Total nuclear extract was then prepared by suspending the pellet in 0.5 ml nuclear lysis buffer (20 mM Tris–HCl [pH 7.5], 150 mM KCl, 3 mM MgCl_2_, 0.3% NP-40 supplemented with 1 complete protease inhibitor cocktail). The input sample was sonicated in a water bath for 15 min while the nuclear fraction and the four washes were sonicated on ice with microtip at 25% duty and 4 output for 60 s each on a Branson ultrasonicator. After sonication, all samples except input were centrifuged at 18,000*g* for 10 min at 4°C. Supernatants were transferred to fresh tubes and used for immunoblotting and RNA isolation. U1 RNA and Lamin A protein were measured as references for the nuclear fraction, and GAPDH mRNA and GAPDH protein were measured as references for the cytoplasmic fraction.

### Immunofluorescence assay and microscopy

1 × 10^5^ cells were seeded in a tissue culture dish with glass bottom (20102018; FluoroDish) and incubated overnight before transfection. Transfections were performed as described above. After transfection, cells were washed three times with ice-cold 1X PBS, then fixed with chilled 100% methanol at room temperature for 5 min, and washed three times with 1X PBS for 5 min. Cells were permeabilized by treatment with 0.2% Triton X-100 for 10 min at room temperature, then washed three times with 1X PBS for 5 min. Then, cells were incubated in 1% BSA (bovine serum albumin) in 1X PBST (1X PBS + 0.1% Tween 20) for 30 min blocking using normal goat serum (ab7481; Abcam) at room temperature. Cells were incubated in primary antibody anti–phospho-histone (γ) H2A.X Ser139 20E3 (60566SF; Cell Signaling Technology, 1:1,000 dilution) for 1 h at room temperature and washed three times with 1X PBS. Then cells were incubated with Goat Anti-Rabbit IgG H&L Secondary Antibody (ab6717; Abcam) for 1 h and washed three times with 1X PBS. Finally, cells were incubated in either 1 μg/ml DAPI DNA stain (4083S; Cell Signaling Technology) or 0.5 μg/ml Hoechst 33342 Reagent (R37165; Thermo Fisher Scientific) for 1 min, rinsed with 1X PBS, and imaged using the Eclipse Ti Inverted Microscope (Nikon) equipped with a 60× objective and Lumencor Sola II LED illumination. Images were captured with a Excelitas pco.edge cMOS camera using Nikon’s NIS-Elements imaging software. Immunofluorescent foci were quantified using ImageJ. Briefly, nuclei were segmented using Li automatic thresholding and converted to a binary mask. Analyze Particles function was used to identify number and area of detected nuclei and create nuclear regions of interest (ROI). Foci within the nuclear boundary of each ROI were identified using Find Maxima with a prominence threshold of >10. Fluorescent images were also analyzed for nuclear morphology. Cells with irregular nuclei (fragmented or lobulated in shape) were analyzed manually and the percentage of cells with damaged nuclei to cells with normal nuclear morphology was calculated for each treatment.

### Protein immunoprecipitation

For each reaction, 3.5 × 10^7^ A549 cells were harvested and resuspended in 1X PBS, then an equal volume of cold polysome lysis buffer was added (20 mM Tris–HCl [pH 7.5], 50 mM KCl, 10 mM MgCl_2_, 1 mM DTT, 1X Complete protease inhibitor cocktail [Roche], 200 U/ml RNase inhibitor murine [New England Biolabs]). Cells were lysed by incubation at 4°C for 5 min followed by incubation at −80°C for about 2 h before centrifuging for 10 min at 20,000*g*. The supernatants were collected and divided so that there were 1 × 10^7^ A549 cells per sample and 5 × 10^6^ cells were saved as whole cell lysate inputs. 75 μl/sample of Dynabeads Protein G (Thermo Fisher Scientific) were washed twice with NT-2 buffer (50 mM Tris–HCl [pH7.5], 150 mM NaCl, 1 mM MgCl_2_, 0.05% NP-40) and resuspended in 100 μl/sample of NT-2 buffer, with 3 μg of each antibody per sample: IgG as a negative control (10284-1-AP; Proteintech), TDP-43 as a positive control (10782-2-AP; Proteintech), and NKAP. The beads/antibody mixtures were incubated for 90 min at room temperature, then beads were washed six times with NT-2 buffer before resuspending in NET-2 buffer (50 mM Tris–HCl [pH 7.5], 150 mM NaCl, 1 mM MgCl_2_, 0.05% NP-40, 20 mM EDTA [pH 8.0], 1 mM DTT, 200 U/ml RNase inhibitor murine). Each antibody had the cell lysate supernatant added so the total volume including the NET-2 buffer would be 1 ml. All three samples were incubated overnight at 4°C with gentle rotation, followed by six washes with NT-2 buffer, and then each sample was split in half for protein and RNA analysis. For RNA samples, the beads were resuspended in NLS elution buffer (20 mM Tris–HCl [pH 8.0], 10 mM EDTA [pH 8.0], 2% NLS, and 2.5 mM TCEP) for proteinase K digest, then the beads were separated and standard RNA silane cleanup with Dynabeads MyOne Silane (Thermo Fisher Scientific) was performed on the supernatant before reverse transcribing the RNA using SuperScript III to perform qRT-PCR. For protein samples, elution 1 was prepared by using 10 μl of 2X Tris-Glycine SDS sample buffer (Thermo Fisher Scientific) to resuspend beads containing immunocomplexes and incubated at 50°C for 10 min, then 9 μl of UltraPure water (Invitrogen) and 1 μl of 0.1 M DTT was added to the elution sample. Elution 2 was collected by resuspending the beads in 10 μl of 2X Tris–Glycine SDS sample buffer and 1 μl of 0.1 M DTT and incubating at room temperature for 5 min before adding 9 μl of UltraPure water to bring the final volume of the sample to 20 μl. All protein samples were heated at 95°C for 10 min before storing at −80°C.

### Cell proliferation assays

The viability of A549, HCT116, and HEK293 cells was determined by modified microculture tetrazolium (MTT) assay (University at Buffalo, State University of New York, NY, USA) or CCK-8 assay (Abcam). Before any treatment, 1.5 × 10^4^ cells were seeded into each well of 96-well flat-bottomed microplates containing medium overnight. The cells were treated and incubated in a humidified incubator in 5% CO_2_ for 48 h at 37°C. For MTT assay, growth medium was replaced with the working MTT solution following the manufacturer’s instructions. The resulting solution was incubated in 5% CO_2_ for another 20 min at 37°C. Formazan crystals were dissolved in 100 μl of DMSO. For CCK-8 assay, 10 μl WST-8 solution was added to the growth medium, and cells were incubated in 5% CO_2_ for 2 h at 37°C. The plates were then analyzed on the Synergy HTX Multi-Mode Microplate Reader (Agilent) at 570 nm. Each sample was assayed with at least three biological replicates.

### Cell cycle analysis with flow cytometry

After treatment, A549 cells were harvested with trypsin and washed twice with 1X PBS. Cells were then permeabilized and fixed with 70% ice-cold ethanol drop-wise and incubated for 15 min on ice, gently vortexing to prevent clumping. Cells were washed twice with 1X PBS. Then, cells were resuspended in PI Staining Solution (3.8 mM sodium citrate, 50 μg/ml PI [Biotium], 40 μg/ml RNase A [Zymo Research]) and incubated for 40 min at 37°C in the dark. Cells were centrifuged and the supernatant was gently aspirated. Finally, the cells were resuspended in 500 μl ice-cold 1X PBS and examined on the S3e Cell Sorter (Bio-Rad Laboratories). The data of cell cycle profiles was analyzed using FlowJo software (BD Biosciences).

### Illumina RNA sequencing

RNA from GRAS1 GapmeR1 knockdown in A549 cells was isolated with RNeasy Mini Kit and purified using RNA Clean & Concentrator Kit. Sequencing was performed for three biological replicates each for both the knockdown and the control samples. Illumina RNA-seq libraries were prepared at the Institution for Genomic Medicine (IGM) using Illumina stranded mRNA prep after poly-A selection. Quality of the libraries was examined by Agilent BioAnalyzer, and sequencing was performed on a NovaSeq S4 with the run type of PE100. Raw paired-end FASTQ sequencing reads were mapped with STAR version 2.7.9a (Dobin et al, 2013) to human reference genome assembly GRCh38 (Schneider et al, 2017) and GENECODE gene annotation file Ensembl 104 (Frankish et al, 2019). FeatureCounts version 2.0.3 (Liao et al, 2014) was used to generate read count matrices, and DESeq2 version 1.34.0 (Love et al, 2014) analysis identified differential expression of RNA transcripts between the triplicate samples of control GapmeR or GRAS1 knockdown GapmeR-treated cells. Gene ontology enrichment analysis was performed with enrichGO version 4.2.2 (Yu et al, 2012). RNA sequencing data are available on the NCBI Sequence Read Archive associated with the BioProject PRJNA979253.

### RNA antisense purification with mass spectrometry

RAP-MS experiments were performed from A549 cells grown in culture. The method was performed as previously described (Trang et al, 2023) with the following modifications. Cells were collected and cross-linked with 254 nm wavelength light using an XL-1500 Spectrolinker (Spectro-UV). Per 20 M cells, the cell pellet was resuspended in 900 μl cold lysis buffer (10 mM Tris–HCl pH7.5, 500 mM LiCl, 0.5% n-dodecyl-β-D-maltoside, 0.2% sodium dodecyl sulfate, 0.1% sodium deoxycholate) with 1X protease inhibitor cocktail and 920 U of murine RNase inhibitor (M0314L; New England Biolabs). Resuspended cell pellets were further lysed on ice by passing through an 18G blunt needle 10 times, followed by two sets of 1.5 min sonication with a Branson sonicator using the microtip (setting: 35 duty, 3.0 power output). Cell lysate was incubated with 20 U of TurboDNase (Invitrogen) for 15 min at 37˚C to remove DNA in the lysate, and then quenched by adding 10 mM EDTA, 5 mM EGTA, and 2.5 mM TCEP. The 6 M urea hybridization buffer was added to cell lysate to a final concentration of 4 M urea, to denature non-specifically interacting proteins. Afterwards, the lysis of 20 M cells was precleared by incubation with 125 μl of MyOne C1 Streptavidin beads (Invitrogen) at 45˚C for 30 min. Biotinylated 90-mer DNA probes for RNA capture (10 μg of probes for GRAS1, 5 μg of probes each for U1 and Luciferase) were mixed with precleared lysate and incubated at 45˚C for 2 h on an Eppendorf ThermoMixer with mixing speed set at 1,000 rpm (with intervals of 15 s shaking, 15 s off). Complexes of biotinylated DNA probes and RNA targets were then captured on MyOne C1 Streptavidin beads by incubating at 45˚C for 1 h with the same mixing setting as above. Beads were washed four times with 4 M urea hybridization buffer, and finally resuspended with 0.5 μl of Benzonase (E1014; Sigma-Aldrich) in 500 μl Benzonase Elution Buffer (20 mM Tris [pH 8.0], 2 mM MgCl2, 0.05% NLS, 0.5 mM TCEP) to elute captured proteins. Proteins were precipitated by adding 80% TCA to final concentration to 20% for overnight incubation at 4˚C. Precipitated proteins were collected by centrifugation for 15 min at 10,000*g* and 4˚C. The protein pellet was resuspended with 50 μl of water containing 5% RapiGest (186002123; Waters) and incubated at 100˚C for 10 min. Next, 0.5 μg of trypsin was added with 37˚C overnight incubation and 600 rpm shaking for protein digestion. Protein samples were purified using HiPPR detergent removal resin (PI88305; VWR International) and dried completely by Speedvac (Savant) before LC-MS/MS analysis at Sanford Burnham Prebys Proteomics Core on a Thermo QExactive instrument. Mass spectrometry datasets as well as MaxQuant peptide search results and parameter files are available via ProteomeXchange with identifier PXD042699.

### Annexin V apoptosis assay

After treatment or transfection, A549 cells were collected by trypsinization and washed in cold 1X PBS. Then cells were resuspended in 100 μl of 1X Annexin-binding buffer (10 mM HEPES adjusted to pH 7.4, 140 mM NaCl, and 2.5 mM CaCl2) with 5 μl of FITC-conjugated Annexin V (A13199; Life Technologies) and 2 μl of 1 mg/ml propidium iodide (40017; Biotium). The cell solution was incubated for 30 mins at room temperature in the dark. After the incubation, 400 μl of 1X Annexin-binding buffer was added to the cell solution and analyzed by the S3e Cell Sorter. The raw data were analyzed using FlowJo software.

## Supplementary Material

Reviewer comments

## Data Availability

The datasets produced in this study are available in the following databases. Illumina high-throughput sequencing run data are available on the NCBI Sequence Read Archive associated with the BioProject PRJNA979253. The mass spectrometry proteomics data have been deposited to the ProteomeXchange Consortium via the PRIDE partner repository with the dataset identifier PXD042699. All unique and stable reagents generated in this study are available from the Lead Contact with a completed Materials Transfer Agreement.
