# Supplementary material for: The noncoding RNA GRAS1 interacts with NKAP and protects against DNA damage and cell death
Source: Life Sci Alliance. 2026 Sep 30;9(12):e202403105. doi: 10.26508/lsa.202403105 (PMC13627766; doi:10.26508/lsa.202403105)
Supplement: Supplementary file 2 [file LSA-2024-03105_TableS1.docx]

**Supplemental Table 1:** Read statistics for RNA-seq samples in this study.

| RNA-seq sample | Read Length | Number of Reads | % Uniquely Mapped |
| --- | --- | --- | --- |
| Control | 101 | 24414878 | 88.74 |
| Control | 101 | 30631523 | 88.85 |
| Control | 101 | 35511447 | 87.32 |
| GRAS1-KD | 101 | 42257830 | 89.39 |
| GRAS1-KD | 101 | 37652306 | 88.91 |
| GRAS1-KD | 101 | 39773180 | 86.26 |
