# Supplementary material for: The noncoding RNA GRAS1 interacts with NKAP and protects against DNA damage and cell death
Source: Life Sci Alliance. 2026 Sep 30;9(12):e202403105. doi: 10.26508/lsa.202403105 (PMC13627766; doi:10.26508/lsa.202403105)
Supplement: Supplementary file 3 [file LSA-2024-03105_TableS2.docx]

**Supplemental Table 2:** Oligonucleotide primer sequences for qRT-PCR and cloning.

| qRT-PCR primers | | |
| --- | --- | --- |
| Target | Forward (5'-3') | Reverse (5'-3') |
| GAPDH | GGGCTCTCCAGAACATCATCC | GTCCACCACTGACACGTTGG |
| TUBB | CCAGACAACTTTGTATTTGGTCAGT | CGTACCACATCCAGGACAGAAT |
| PML | CCGTCATAGGAAGTGAGGGCTTC | GTTTTCGGCATCTGAGTCTTCCG |
| NKAP | GCTGGAAAACGTATCCCACGAAG | CTGGTTCTCTTTTCGCAGTCGC |
| PLK3 | CTATGTGGCTCCAGAAGTGCTG | TCCTTCAGGTCAGCCGTCTCAA |
| GADD45a | TGCTCAGCAAAGCCCTGAGT | GCAGGCACAACACCACGTTA |
| U1 | TTACCTGGCAGGGGAGATAC | TCCCACATTTGGGGAAATC |
| GRAS1 | CCTTTTGGTGCGAGCTTGCTGT | GTCGACTCACCAGACTCAGGGTG |
| SNORA13 | AGCCTTTGTGTTGCCCATTC | AACTGTTACTTATGCAGCTCCT |
| Cloning primers | | |
| Target | Forward (5'-3') | Reverse (5'-3') |
| GRAS1 | CCTTTTGGTGCGAGCTTGC | GAGGCATCACTTTCAGACTGC |
| NKAP | ATGGCTCCGGTGTCCGG | TTATTTGTCATCCTTCCCTTTGGTCT |
| SNORA13 | AGCCTTTGTGTTGCCCATTC | AACTGTTACTTATGCAGCTCCT |
| TIGA1 | ATGGTACAGCCCGCTC | TTATCTGGAGAACTCCCTGG |
